# Visual Scanning Patterns during the Dimensional Change Card Sorting Task in Children with Autism Spectrum Disorder

**DOI:** 10.1155/2012/123053

**Published:** 2012-09-20

**Authors:** Li Yi, Yubing Liu, Yunyi Li, Yuebo Fan, Dan Huang, Dingguo Gao

**Affiliations:** ^1^Department of Psychology, Sun Yat-sen University, 135 Xingang West Road, Guangzhou, Guangdong 510275, China; ^2^Guangzhou Cana School, Longxing Middle Rd, Guangzhou, Guangdong 510540, China; ^3^Guangzhou Rehabilitation & Research Center for Children with ASD, Longxing Middle Rd, Guangzhou, Guangdong 510540, China

## Abstract

Impaired cognitive flexibility in children with autism spectrum disorder (ASD) has been reported in previous literature. The present study explored ASD children's visual scanning patterns during the Dimensional Change Card Sorting (DCCS) task using eye-tracking technique. ASD and typical developing (TD) children completed the standardized DCCS procedure on the computer while their eye movements were tracked. Behavioral results confirmed previous findings on ASD children's deficits in executive function. ASD children's visual scanning patterns also showed some specific underlying processes in the DCCS task compared to TD children. For example, ASD children looked shorter at the correct card in the postswitch phase and spent longer time at blank areas than TD children did. ASD children did not show a bias to the color dimension as TD children did. The correlations between the behavioral performance and eye moments were also discussed.

## 1. Introduction

Executive dysfunction has been well studied and consistently found in previous studies in children with autism spectrum disorder (ASD) [1–3]. Executive function (EF) refers to a wide range of abilities including planning, inhibition, working memory, cognitive flexibility, impulse control, and so forth [1, 2, 4, 5]. The EF deficit in ASD has been found to be related to their symptoms of restricted and repetitive behaviors [6, 7], and their impairments in Theory of Mind [8–12]. 

Impaired EF has been found in ASD children in numerous previous studies, including the impaired planning skills in the Tower of Hanoi or the Tower of London task [13–17], the impaired visual spatial working memory [18–28] and verbal working memory [23, 29], the impaired inhibitory control in the Stroop task [20, 30, 31], and the deficit in cognitive flexibility in the Wisconsin Card Sorting Task (WCST) [13, 32]. However, contradictory findings came from studies that did not find EF deficits in ASD, for example, in the working memory task [30] and in the WCST [31].

The Dimensional Change Card Sorting task, developed by Frye et al. [33], was widely used to measure the cognitive flexibility of typically developing (TD) preschoolers. Compared with the WCST, the DCCS task is relatively simple and suitable to use across a wide age range [34]. The DCCS task asks children to sort bidimensional test cards (e.g., a red rabbit and a blue boat) with one of the two target cards (e.g., a red boat and a blue rabbit) according to one rule (e.g., by color), and then after several successful trials, switch to another rule (e.g., by shape). Previous findings using the DCCS task indicated that most 3-year olds had difficulty switching between the dimensions, but from age 4, most children could successfully switched to the new rule [35, 36]. Cognitive flexibility continues to improve beyond the preschool years up to age 7, reflected in their performance in the Advanced DCCS task [36], also referred to as the border version of the DCCS task [34]. The Advanced DCCS task presents the rule with a visual cue (e.g., a border around the object or a star on the card) and requires children to switch between the two dimensions back and forth according to the visual cue (e.g., sorting by color when there is a border, and by shape when there is no border) [37, 38].

The present study used a computerized DCCS task, including the standard and the border versions [34], to examine ASD children's deficits in cognitive flexibility. Cognitive flexibility is required in the DCCS task to shift to the action of sorting the cards according to the alternative dimension from the previous dimension. The cognitive flexibility of ASD children had been assessed with DCCS task in previous studies [9, 11, 12, 39]. The study by Ditcher and colleagues reported significantly slower and less accurate performance of ASD children in the computerized DCCS task compared to TD children [39]. This finding confirmed previous findings of EF deficits in ASD children using other EF tasks. Zelazo and his colleagues tested ASD children in the DCCS task and the Theory of Mind task and found the correlation between the performance in the two tasks in children with high-functioning ASD but not in those with low-functioning ASD [12].

In addition to the behavioral measures, we also used eye-tracking technique to explore ASD children's visual scanning patterns during the computerized DCCS task. Eye-tracking technique allowed us to examine children's ability to strategically allocate attention while looking at stimuli, and thus to explore ASD children's deficit in the cognitive flexibility during the DCCS task. The eye-tracking technique could be used to explore the cognitive flexibility in the DCCS task because it can reflect how much difficulty children experiences when they shift from one dimension to the other. Minar tracked children's eye movements during the DCCS task and demonstrated that 3-year olds who passed preswitch phase of the DCCS task could focus their visual attention on relevant dimensions of the stimuli for longer length of time than those who failed. It suggested that young children who failed in the DCCS task may have trouble shifting their visual attention away from irrelevant features of the stimuli or nonrelevant sorting dimensions [40]. Chevalier et al. also reported that in the Advanced DCCS task, younger TD children showed more difficulty inhibiting irrelevant information compared to older ones [41].

**Figure 1:**
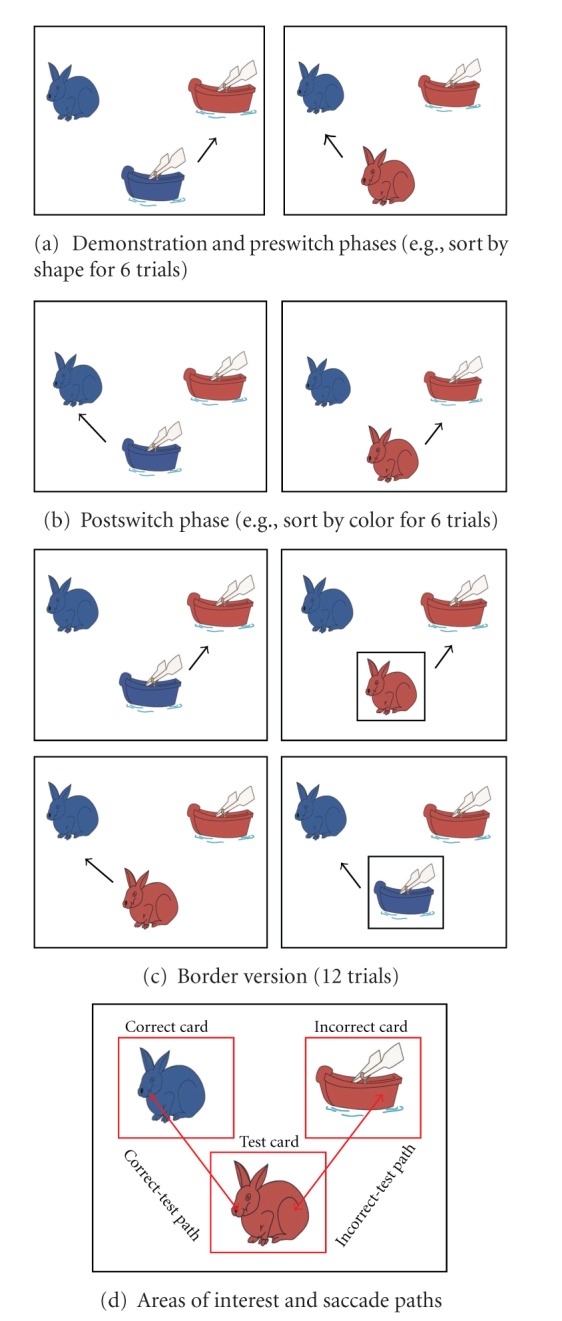
Illustration of trials in the preswitch phase (a), postswitch phase (b), and the border version (c), and examples of areas of interest (AOI) and saccade paths (d). Upper cards were targets and the bottom card was the test card. Arrows show correct sorts (pictures from [34]).

The current study also used eye-tracking technique to examine whether ASD children showed different visual scanning patterns from TD children, which may reflect their difficulty in inhibiting irrelevant information in the DCCS task. In order to do this, we used two approaches to analyze the eye-tracking data. First, we investigated how children's attention was distributed on the computer screen when they were presented the target cards at the upper field and the test card at the bottom (Figure 1). Using a traditional areas-of-interest (AOI) approach, the fixation durations within each card were calculated and compared between subject groups and cards. Second, to examine how attention was shifted from one card to another, we analyzed children's saccade paths between cards, especially the path between the correct and the test cards, and the one between the incorrect and the test cards. The frequencies of the two scanpaths were calculated and compared between the groups. Finally, children's behavioral performance in the DCCS task was correlated to their eye movement patterns.

On the basis of previous findings on EF deficits in ASD, we expected poorer behavioral performance in the DCCS task in ASD children than TD children. We also expected atypical visual scanning patterns in ASD children compared with TD children. Chevalier et al. [41] reported an asymmetry in fixation durations between the two dimensions; since color matching was reported to be easier than shape matching [42, 43], children experienced more difficulty inhibiting color than shape, and thus fixated longer when inhibiting color than inhibiting shape. We predicted similar patterns in TD children but the pattern for ASD children was unpredictable.

## 2. Method

### 2.1. Participants

Participants were 18 ASD children and 31 TD children matched by their verbal mental age (VMA), which was measured by the Chinese version of Peabody Picture Vocabulary Test (PPVT) [44] (Table 1). TD children were recruited from normal preschools in Guangzhou, China. ASD children were recruited from a special school for ASD children in the same city. See Table 1 for details of the participants. All ASD children were previously diagnosed by clinicians and satisfied the diagnostic criteria for autism according to the DSM-IV [45]. Diagnosis of ASD children was confirmed using the Chinese version of the Autism Spectrum Quotient: Children's Version (AQ-Child) [46]. Mean AQ scores of ASD children were significantly above the cut-off score (76), *t*(16) = 4.99, *P* < .001. There was no group difference of VMA, *t*(45) = 0.062. 

### 2.2. Materials and Procedure

Each child was tested individually in a quiet room. We used the same material and procedure of the DCCS task following the standardized protocol described by Zelazo [34], including the demonstration phase, the preswitch phase, the postswitch phase, and the border version. Children were asked to play a sorting game (either a “color” or a “shape” game). In each trial children were shown a bivalent test card (the bottom one) and two bivalent target cards (the upper ones) with two dimensions (i.e., color and shape). For example, as shown in Figure 1, the target cards were a blue rabbit and a red boat, and the test card could be a blue boat or a red rabbit. Children were asked to sort the test card with one of the target cards according to a certain rule (e.g., color or shape). Children were asked to point out which target card they would like to place with the test card. Their choice was manually recorded in an answer sheet and their eye movements were recorded by a Tobii T60 eye tracker during the whole procedure. After they made the choice, the experimenter pressed a button to go to the next trial, until they finished all trials in the phase.

The demonstration phase used real cards, and from the preswitch phase, the cards were displayed on a computer screen with a resolution of 1024 × 768 pixels connected to the eye tracker. Before eye-tracking, children received a calibration in the Tobii Studio calibration program, which asked children to follow a toy duck with their eyes as it bouncing around the screen. Calibrations were considered successful when all 5 points showed good fit in the computed mapping for both eyes. In the case of binocular vision differences, 5 good-fit points for a single eye were also accepted.

#### 2.2.1. Demonstration Phase

The demonstration phase was a practice for children to familiarize the rules. It included four trials using real cards. Children were asked to recognize the color (i.e., blue and red) and the identity (i.e., a boat or a rabbit) of the content of the target cards first. The experimenter demonstrated the procedure of sorting by the color or the shape dimension (depending on the order the child was assigned in). Then children were asked to do the same thing on their own. Feedbacks were given to make sure children understood and sorted by the rule. Children were randomly assigned into two orders: the rule for Order 1 was to sort by color first, and the rule for Order 2 was to sort by shape first. 

#### 2.2.2. Preswitch Phase

The preswitch phase contained six trials. In each trial, the child was asked to choose a target card they would like to place with the test card. The test cards appeared in a random order. No feedback was provided after they made the choice. If children were correct in at least 5 out of 6 trials, they passed this phrase and proceeded to the postswitch phase; if not, they would stop here. Children who passed the preswitch phase were scored 1 and those who did not pass the preswitch phase were scored 0.

#### 2.2.3. Postswitch Phase

Children were asked to change the rules in the postswitch phase. “We're not going to play the color game anymore. Now we are going to play a new game.” Then an alternative rule was explained to the child. For children in Order 1, the rule was changed to “sorting by shape” and for children in Order 2, the rule was changed to “sorting by color”. The postswitch phase also contained 6 trials where the two test cards appeared in a random order. Children who passed this phase by being correct in at least 5 out of 6 trials in the postswitch phase were scored 2 and preceded to the border version. 

#### 2.2.4. Border Version

The border version used the same target cards as in the previous phases. There were four types of test cards that appeared in a random order, as shown in Figure 1(c), a blue boat, a red rabbit, a blue boat with a black border, and a red rabbit with a black border. Children were instructed to sort the border test cards by color, and the cards without the border by shape, which was the same for both orders. Children passed the border version and scored 3 when they were correct in at least 9 out of 12 trials.

## 3. Results

### 3.1. DCCS Scores

All 31 TD children and 17 out of 18 (94.44%) ASD children passed the preswitch phase and scored 1; 22 or 70.97% TD children and 8 or 44.44% ASD children passed the postswitch phase and scored 2; 11 or 35.48% TD children and 2 or 11.76% ASD children passed the border version and scored 3. An independent *t* test was conducted to test the group difference of the DCCS scores. Results showed that TD children (*M* = 2.06, *SD* = 0.81) got significantly higher DCCS scores than ASD children (*M* = 1.50, *SD* = 0.79), *t*(48) = −2.37, *P* = .022, *η* ^2^ = .11.

### 3.2. Eye Movements

In each phase and group, outliers of the total fixation durations of the whole picture (including the 3 cards and the blank area) were removed from further analyses (3 standard deviations beyond the mean, about 2.28% of the data points). To test the group difference of looking time spent on the whole picture, we conducted independent *t* tests for each phase. Results showed no group difference of total looking time in the preswitch phase, *t*(47) = −0.64, *P* = .53, *η* ^2^ = .009, in the postswitch phase, *t*(19.33) = 0.86, *P* = .40, *η* ^2^ = .037, or in the border version, *t*(28) = −1.20, *P* = .24, *η* ^2^ = .049.

**Figure 2:**
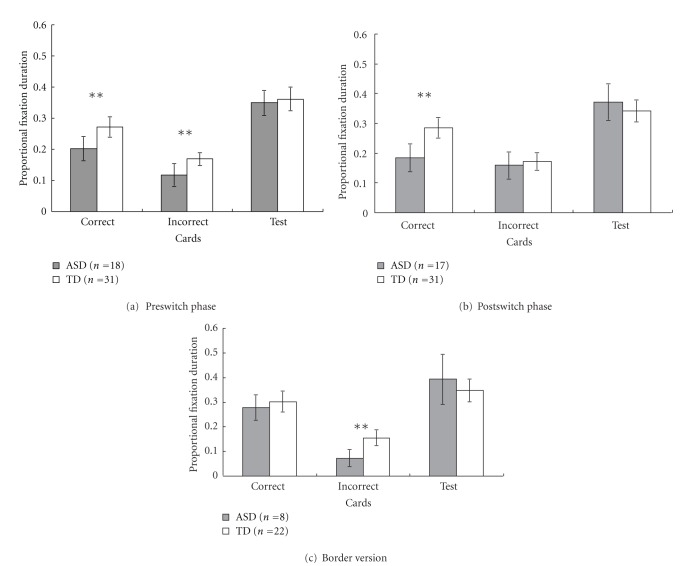
Proportional fixation durations within the correct, incorrect, and the test cards for ASD and TD children during the preswitch phase (a), the postswitch phase (b), and the border version (c). (Error bars represent 95% confidence intervals). **P < .01.

We employed two approaches to analyze the eye movement data. The first approach was the traditional AOI approach, which predefined AOIs including the test card and the two target cards (one as the correct card, the other as the incorrect card, according to the rule). The AOIs were defined as the entire face feature of interest plus an additional 50 pixels of edges. All fixation durations falling within each AOI for each trial were summed up to calculate total fixation durations. Considering the variability of children's total looking time in each trial, we calculated the proportional fixation durations by dividing the total fixation time on each AOI by the total fixation time on the whole picture. Results of the analysis of proportional fixation durations for ASD and TD children in each phase were shown in Figure 2.

**Figure 3:**
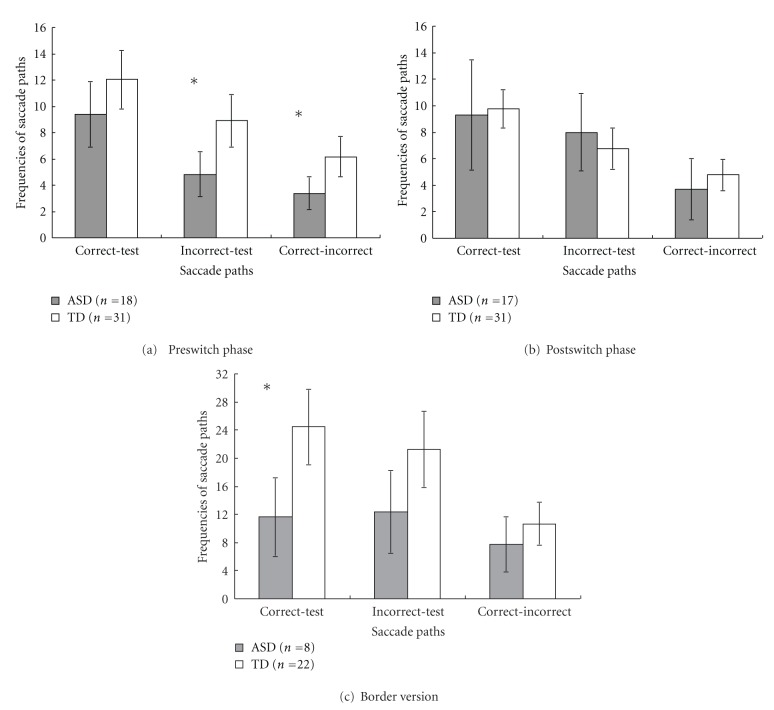
Frequencies of the correct-test, the incorrect-test, and the correct-incorrect paths for ASD and TD children during the preswitch phase (a), the postswitch phase (b), and the border version (c). (Error bars represent 95% confidence intervals). *P < .05.

The second approach was the analysis of saccade paths, which counted the frequencies of a participant's gaze shifts from one AOI to another. We calculated the frequencies of three saccade paths involving the path between the correct and the test cards (correct-test path), the path between the incorrect and the test cards (incorrect-test path), and the path between the correct and the incorrect cards (correct-incorrect path). Results of saccade path analysis for ASD and TD children in each phase were shown in Figure 3. 

Firstly, Because of highly similar data patterns for fixations in the two orders and the limited sample size, we combined all fixations of participants in both orders for subsequent data analysis. We did the AOI and the scanpath analyses for the preswitch phase, the postswitch phase, and the border version separately. Then, we conducted an exploratory analysis to compare children who passed and failed in the postswitch phase, and children in Order 1 and 2 in the postswitch phase. Last, we correlated children's DCCS scores with their eye movements. 

#### 3.2.1. Proportional Fixation Duration

To test the difference in looking time spent on each AOI between ASD and TD children, we conducted 2 (Subject Group: ASD versus TD) × 3 (AOI: correct, incorrect, and test cards) mixed-design ANOVAs for each phase. Results were shown in Figure 2(a). In the preswitch phase, there was a significant effect of Subject Group, *F*(1, 47) = 18.87, *P* < .001, *η* ^2^ = .29, and a significant AOI effect, *F*(2, 94) = 56.35, *P* < .001, *η* ^2^ = .55. No Group × AOI interaction was found, *F*(2, 94) = 1.05, *P* = .36, *η* ^2^ = .022. Pairwise comparison *t* tests were obtained to compare mean fixation durations between groups for each of the three AOIs, and then to compare fixation durations between AOIs for each group. Results showed that compared to ASD children, TD children looked longer at the correct card, *t*(47) = −2.72, *P* = .009, *η* ^2^ = .14, and the incorrect card, *t*(47) = −2.61, *P* = .012, *η* ^2^ = .13. For each group, children spent longer looking time on the correct card than the incorrect card, *p*s < .001, and longer looking time on the test card than the correct and the incorrect cards, *p*s < .01. A pairwise *t* test was also conducted to compare the ASD and TD children's proportional fixation duration at the blank area. Results showed that ASD children looked longer at the blank area (*M* = .33, *SD *= .13) than TD children (*M* = .20, *SD* = .08), *t*(47) = 4.34, *P* < .001, *η* ^2^ = .29. 

In the postswitch phase, a 2 (Subject Group) × 3 (AOI) ANOVA was found a significant effect of Subject Group, *F*(1, 46) = 6.70, *P* = .013, *η* ^2^ = .13, a significant AOI effect, *F*(2, 92) = 33.02, *P* < .001, *η* ^2^ = .42, and the Group × AOI interaction, *F*(2, 92) = 3.88, *P* = .024, *η* ^2^ = .078. Pairwise comparison *t* tests showed that TD children spent longer looking time at the correct card than ASD children, *t*(46) = −3.45, *P* = .001, *η* ^2^ = .21. TD children looked longer at the correct card than the incorrect card, *t*(30) = 4.33 *P* < .001, *η* ^2^ = .38. There was no difference of looking time spent on the correct and the incorrect cards for ASD children, *t*(16) = 0.67, *P* = .52, *η* ^2^ = .027 (Figure 2(b)). A pairwise *t* test showed longer proportional fixation durations at the blank area in ASD (*M* = .28, *SD* = .10) than TD children (*M* = .20, *SD* = .11), *t*(46) = 2.59, *P* = .013, *η* ^2^ = .13.

In the border version, a 2 (Subject Group) × 3 (AOI) ANOVA found a significant AOI effect, *F*(2, 56) = 34.707, *P* < .001, *η* ^2^ = .55. There was no Group effect and Group × AOI interaction, *F*(1, 28) = 1.58, *P* = .22, *η* ^2^ = .053; *F*(2, 56) = 2.09, *P* = .13, *η* ^2^ = .069. Pairwise comparison *t* tests showed that TD children spent longer time looking at the correct card than the incorrect card, *t*(21) = 4.73, *P* < .001, *η* ^2^ = .52. ASD children also spent longer time looking at the correct than the incorrect cards, *t*(7) = 5.55, *P* = .001, *η* ^2^ = .81. TD children spent longer time than ASD children in looking at the incorrect card, *t*(28) = −2.82, *P* = .009, *η* ^2^ = .22 (Figure 2(c)). A pairwise *t* test showed longer proportional fixation durations at the blank area in ASD (*M* = .30, *SD* = .15) than TD children (*M* = .17, *SD* = .07), *t*(28) = 3.16, *P* = .004, *η* ^2^ = .26.

#### 3.2.2. Analysis of Saccade Paths

Three saccade paths, the correct-test path, the incorrect-test path, and the correct-incorrect path, were identified and their frequencies were calculated for each phase and group. A 2 (Subject Group: ASD versus TD) × 3 (Path Type: correct-test, incorrect-test, and correct-incorrect paths) mixed-design ANOVA was conducted to test the differences of frequencies of saccade paths between groups and types of paths. Results were shown in Figure 3. In the preswitch phase, there was a significant effect of Path Type, *F*(2, 94) = 36.43, *P* < .001, *η* ^2^ = .44, and a significant effect of the group, *F*(1, 47) = 6.69, *P* = .013, *η* ^2^ = .13. No Group × Path Type interaction was found, *F*(2, 94) = 0.63, *P* = .54, *η* ^2^ = .013. Pairwise comparison *t* tests were run to compare mean path frequencies between groups for each path, and then between paths for each group. Results showed that compared to TD children, ASD children scanned less often between the incorrect card and the test card,* t*(47) = −2.77, *P* = .008, *η* ^2^ = .14, and between the correct and the incorrect card, *t*(47) = −2.49, *P* = .016, *η* ^2^ = .12. ASD and TD children both scanned between the correct and the test cards more than between the incorrect and the test cards, between the correct and the incorrect cards, *p*s < .05. Both groups also scanned between the incorrect and the test cards more often than between the correct and the incorrect cards, *p*s < .05 (Figure 3(a)).

In the postswitch phase, a 2 (Subject Group) × 3 (Path Type) mixed-design ANOVA found a significant effect of Path Type on the frequencies of paths, *F*(2, 46) = 27.41, *P* < .001, *η* ^2^ = .37. There was no effect of Subject Group, *F*(1, 46) = 0.005, *P* = .95, *η* ^2^ = .000, or the Group × Path Type interaction, *F*(2, 46) = 1.41, *P* = .25, *η* ^2^ = .030. Pairwise comparison *t* tests found no group difference in either the correct-test or the incorrect-test paths, *p*s > .05. TD children scanned between the correct and the test cards more often than between the incorrect and the test cards, *t*(30) = 3.92, *P* < .001, *η* ^2^ = .34. ASD children showed no differences between the frequencies of these two paths, *t*(16) = 0.69, *P* = .50, *η* ^2^ = .029. Both groups scanned between the correct and the test cards, and between the incorrect and the test cards more often than they scanned between the correct and the incorrect cards, *p*s < .05 (Figure 3(b)).

In the border version, a 2 (Subject Group) × 3 (Path Type) mixed-design ANOVA found a Group effect on the frequencies of paths, *F*(1, 28) = 5.03, *P* = .033, *η* ^2^ = .15, and a significant effect of Path Type, *F*(2, 56) = 12.97, *P* < .001, *η* ^2^ = .32, and a Group × Path Type interaction, *F*(2, 56) = 3.52, *P* = .036, *η* ^2^ = .11. Pairwise comparison *t* tests found that TD children scanned between the correct and the test cards more often than ASD children, *t*(28) = −2.67, *P* = .012, *η* ^2^ = .20. TD children scanned following the correct-test path and the incorrect-test path more often than the correct-incorrect path, *t*(21) = 6.15, *P* < .001, *η* ^2^ = .64; *t*(21) = 4.96, *P* < .001, *η* ^2^ = .53. ASD children scanned between the three type of paths similarly, *p*s > .05 (Figure 3(c)).

#### 3.2.3. An Exploratory Analysis: Pass versus Fail

**Figure 4:**
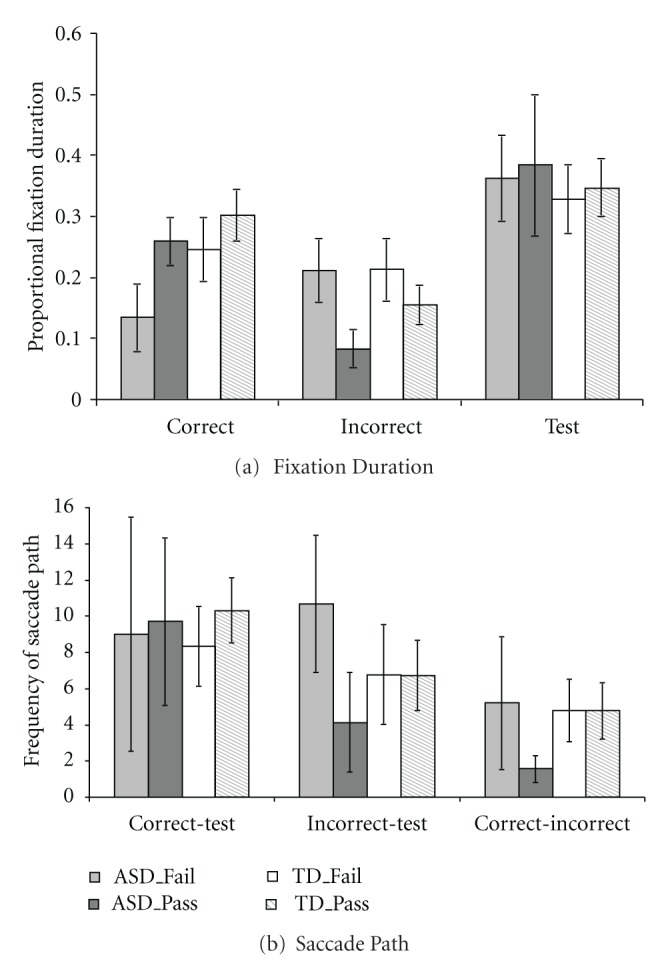
Proportional fixation durations within the correct, incorrect, and the test cards (a) and frequencies of the correct-test path, the incorrect-test path, and the correct-incorrect path (b) for ASD and TD children who passed and failed in the postswitch phase. (Error bars represent 95% confidence intervals).

There are 22 TD and 8 ASD children who passed postswitch phase, while 9 TD and 9 ASD children failed in the postswitch phase. Due to the limited sample sizes in each group, we conducted an exploratory analysis in order to investigate the difference of visual scanning patterns between these children who passed and those who failed. We created 4 groups (i.e., ASD_Pass, ASD_Fail versus TD_Pass versus TD_Fail) and ran a 4 (Group) × 3 (AOI) mixed-design ANOVA. This analysis was only conducted for the postswitch phase, due to the limited sample sizes for children who failed in the preswitch phase and ASD children who passed the border version. Results, as shown in Figure 4(a), showed a significant AOI effect, *F*(2, 88) = 33.57, *P* < .001, *η* ^2^ = .43, and a significant AOI × Group interaction, *F*(6, 88) = 4.15, *P* = .001, *η* ^2^ = .22. There was no effect of the group, *F*(3, 44) = 2.25, *P* = .096, *η* ^2^ = .13. Post hoc analysis with the Tukey HSD method showed that ASD children who failed in the postswitch phase looked at the correct card shorter than ASD children who passed, and both groups of TD children, *p*s < .05; ASD children who passed the postswitch phase looked at the incorrect card shorter than ASD children who failed and TD children who failed, *p*s < .05. Pairwise comparison *t* tests further found both ASD and TD children who passed the postswitch phase showed similar visual scanning patterns; they both looked longer at the correct card than the incorrect card,* t*(6) = 6.77, *P* = .001, *η* ^2^ = .88, *t*(21) = 4.73, *P* < .001, *η* ^2^ = .52, respectively. For ASD and TD children who failed in the postswitch phase, there was no difference of looking time at the correct card and the incorrect card, *t*(9) = −1.95, *P* = .083, *η* ^2^ = .30, *t*(8) = 0.83, *P* = .43, *η* ^2^ = .080, respectively.

A 4 (Group: ASD_Pass, ASD_Fail versus TD_Pass versus TD_Fail) × 3 (Path Type: correct-test path, incorrect-test path versus correct-incorrect path) mixed-design ANOVA found a significant effect of Path Type, *F*(2, 44) = 26.96, *P* < .001, *η* ^2^ = .38, and a significant Path Type × Group interaction, *F*(6, 44) = 2.57, *P* = .024, *η* ^2^ = .15. There was no effect of the group, *F*(3, 44) = 0.83, *P* = .49, *η* ^2^ = .052. Post hoc analysis with the Tukey HSD method showed that ASD children who failed in the postswitch phase scanned between the incorrect card and the test card more often than ASD children who passed, *P* = .033 (Figure 4(b)).

#### 3.2.4. The Effect of Dimensions

**Figure 5:**
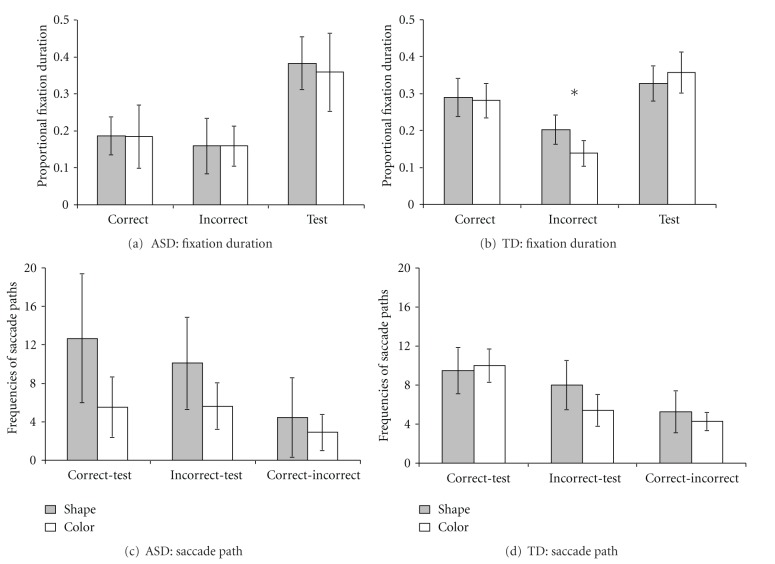
Proportional fixation durations within the correct, incorrect and the test cards for ASD (a) and TD children (b) following the shape rule (Order 1) or the color rule (Order 2) in the postswitch phase. (Error bars represent 95% confidence intervals). *P < .05.

The effect of dimensions was investigated using a 2 (Dimension: sorting by color or shape) × 2 (Subject Group: ASD versus TD) × 3 (AOI: correct, incorrect, and test cards) mixed design ANOVA. Results showed a significant effect of the Subject Group, *F*(1, 44) = 6.62, *P* = .014, *η* ^2^ = .13, a significant AOI effect, *F*(2, 88) = 32.62, *P* < .001, *η* ^2^ = .43, and the Group × AOI interaction, *F*(2, 88) = 3.78, *P* = .027, *η* ^2^ = .08. No significant main effect of Dimension or interaction involving Dimension was found, *p*s > .05. Pairwise comparison *t* tests showed that TD children following the shape rule spent longer looking time at the incorrect card than those TD children following the color rule, *t*(29) = 2.38, *P* = .024, *η* ^2^ = .21. This indicated that it was harder for TD children to inhibit the color dimension than to inhibit the shape dimension. No difference of dimension was found for ASD children or in other AOIs for TD children, *ps* > .05 (Figures 5(a) and 5(b)).

A 2 (Dimension) × 2 (Subject Group) × 3 (Path Type) mixed-design ANOVA found a significant effect of Path Type on the frequencies of paths, *F*(2, 44) = 28.26, *P* < .001, *η* ^2^ = .39, and a significant Dimension effect, *F*(1, 44) = 4.90, *P* = .032, *η* ^2^ = .10. There was no effect of Subject Group, *F*(1, 44) = 0.026, *P* = .87, *η* ^2^ = .001, or the Group × Path Type interaction, *F*(2, 44) = 1.49, *P* = .23, *η* ^2^ = .033. Pairwise comparison *t* tests found no difference of dimensions in either path for ASD and TD children, *p*s > .05 (Figures 5(c) and 5(d)).

### 3.3. Correlations

**Figure 6:**
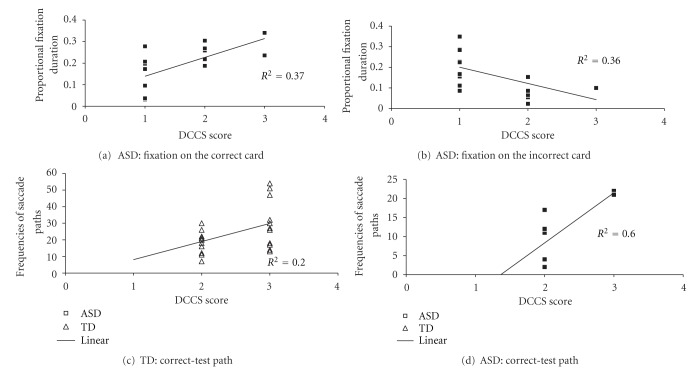
Correlations of DCCS scores with, ASD children's proportional fixation duration on the correct card in the postswitch phase (a), ASD children's proportional fixation duration on the incorrect card in the postswitch phase (b), TD children's frequency of the correct-test path in the border version (c), and ASD children's frequency of the correct-test path in the border version (d).

Pearson correlation coefficients were calculated to measure the correlationsbetween DCCS scores and children's ages, VMA, and eye movement index, including proportional fixation durations and frequencies of saccade paths, for ASD and TD children separately. Results, as shown in Figure 6, showed that DCCS scores were positively correlated with age in TD children, *r*_*p*_(31) = .43, *P* = .016, but not ASD children, *r*_*p*_(18) = −.033, *P* = .90. There was no correlation between VMA and DCCS scores for ASD or TD children, *r*_*p*_(18) = .17, *P* = .49, *r*_*p*_(31) = .31, *P* = .093, respectively. The DCCS scores and the eye movements during the DCCS task were correlated in several ways. For TD children, DCCS scores were positively correlated with the frequencies of the correct-test path in the border version, *r*_*p*_(22) = .44, *P* = .039. For ASD children, DCCS scores were positively correlated with proportional fixation duration on the correct card in the postswitch phase, *r*_*p*_(17) = .65, *P* = .005, and the frequencies of correct-test path in the border version, *r*_*p*_(8) = .77, *P* = .024. ASD children's DCCS scores were also negatively correlated with proportional fixation durations on the incorrect card in the postswitch phase, *r*_*p*_(17) = −.60, *P* = .010. 

## 4. Discussion

The present study explored the visual scanning patterns to reveal the underlying processes for ASD and TD children during the DCCS task. The behavioral performance of ASD children in the DCCS task was significantly poorer than TD children, which was consistent with previous findings on EF deficits in ASD children using behavioral measurements, particularly the DCCS task (e.g., [9, 11, 12, 39]). In addition to the behavioral performance in the DCCS task in ASD children, the current study further investigated their visual scanning patterns in the DCCS task using eye tracking. 

Our analysis of eye movements mainly focused on the postswitch phase, which required children to switch their acquired rule in the preswitch phase to an alternative rule. Results from the analysis of proportional fixation durations revealed atypical visual scanning patterns in ASD children during the postswitch phase in the DCCS task compared to TD children; TD children looked longer at the correct card than the incorrect card, whereas ASD children spent similar amount of time at the correct and the incorrect card. We did not find longer looking time in the irrelevant information in ASD children than TD children. Instead, the proportional duration in the relevant information was shorter for ASD than TD children (see Figure 2(b)). A reasonable interpretation was that ASD children spent more time on blank areas of the screen (i.e., areas other than the three cards) than TD children. This account was supported by the findings of *t* tests comparing ASD and TD children's proportional fixation durations spent on the blank area. This finding was consistent with previous findings on ASD children's tendency to attend to background information which revealed their impairments in information processing (e.g., [47, 48]). 

When we examined difference of visual scanning patterns between the children who passed and those who failed in the postswitch phase, both ASD and TD children who passed the postswitch task looked longer at the correct than the incorrect cards, but those who failed spent similar amount of time looking at the correct and the incorrect cards (Figure 4(a)). This result implied that for those who failed in this task, the underlying process may be similar for ASD children and TD children, although ASD children who failed spent less looking time at the correct card than TD children who failed. The analysis of saccade paths showed that ASD children who failed in the postswitch phase scanned more often between the incorrect and the test card than those who passed (Figure 4(b)). This may reveal the impairment in some ASD children to inhibit the acquired rule and to switch to a new one. However, due to the limited sample sizes of each group and their considerable variations, these findings may not be conclusive without further evidence. 

Chevalier et al. [41] found that in the DCCS task, inhibiting color was harder and thus triggered longer fixation time than inhibiting shape. Our data with TD children was consistent with this conclusion by showing that TD children spent longer fixation time looking at the incorrect card when inhibiting color than inhibiting shape. That is, when the rule was sorting by shape and inhibiting color, TD children looked longer at the irrelevant information more than when the rule was sorting by color and inhibiting shape. This result confirmed that inhibiting color was more difficult than inhibiting shape for TD children, but not for ASD children, who spent similar amount of time across dimensions (Figure 5(a)). The most straightforward interoperation was that ASD children did not show a bias to the color dimensions as TD children did. This account needs further evidence with sophisticated experimental design to explore the effect of the dimension. 

In the border version, which required higher-level cognitive flexibility, ASD children spent similar amount of time looking at all three cards as TD children (Figure 2(c)). Analysis of saccade path found that ASD children scanned less often between the correct and the test cards in the border version (Figure 3(c)). This result may indicate that in this advanced version of the DCCS task, ASD children were less likely to build connections between the correct target card and the test card, although they looked long enough at each of the cards. 

The DCCS scores were correlated with ages of TD children, which confirmed previous findings on the development of DCCS performance in preschool years [34]. However, ASD children's ages were not correlated with their DCCS scores. This may be because of different ages of the two groups; we examined TD children between 3- to 7-year-olds with a mean age of 6, and ASD children with a wider age range (3- to 9-years old) with a mean age of around 4. In addition, matching on VMA may underestimate the abilities of children with ASD, which may be responsible for the lack of correlation [11, 49]. 

The DCCS scores were positively correlated with the frequencies of paths between the correct card and the test card in the border version for both ASD and TD children. This finding indicated that the more often ASD and TD children scanned between the correct and the test cards, the more accurate they were in their behavioral performance in the DCCS task. For ASD children, the DCCS performance was positively correlated with their fixation time on the correct card, and negatively correlated with their fixation time on the incorrect card. This indicated that the better ASD children were at inhibiting irrelevant information and allocating their attention at the relevant information, the more accurate they were in the DCCS task performance. 

The present study had some limitations. For example, the limited sample size in certain groups (e.g., the Fail Group in the preswitch phase and the Pass Group in the border version) made it difficult to perform certain analysis (e.g., to compare the Pass and Fail Groups in these two conditions). Future research should use samples of ASD and TD children with larger sizes to gain richer data. Future research could also explore how the visual scanning patterns in the DCCS task correlates to other EF performance, and other abilities such as Theory of Mind. Moreover, as we discussed before, a well-designed research could be conducted to explore the effect of the dimension in the DCCS task in ASD children.

## 5. Conclusions

The purpose of the present study was to explore the visual scanning patterns in the DCCS task in ASD children. We used two data analysis approaches, the traditional AOI approach and the analysis of saccade path, to analyze the eye movements of ASD and TD children in the DCCS task. Results support ASD children's impairments in cognitive flexibility in behavioral measurements. ASD children also show some atypical visual scanning patterns in the DCCS task compared to TD children. The visual scanning patterns, especially the proportional fixation durations and the frequencies of saccade paths, are correlated with children's behavioral performance in the DCCS task. 

## Figures and Tables

**Table 1:** Participant characteristics in each group.

Variable	ASD	TD	Group difference
Male (female)	17 (1)	27 (4)	Fisher's exact test, *P* = .64
Age range	3.75–9.58	3.00–6.92	—
Mean age	6.61 (1.46)	4.37 (1.09)	*t*(47) = 6.11, *P* < .001
VMA	37.5 (30.64)	38 (24.34)	*t*(45) = −0.062, *P* = .95
AQ	88.29 (10.17)	—	—

Standard deviations are shown in parentheses.
